# Impact of yoga on blood pressure and quality of life in patients with hypertension – a controlled trial in primary care, matched for systolic blood pressure

**DOI:** 10.1186/1471-2261-13-111

**Published:** 2013-12-07

**Authors:** Moa Wolff, Kristina Sundquist, Sara Larsson Lönn, Patrik Midlöv

**Affiliations:** 1 Center for Primary Health Care Research, Department of Clinical Sciences in Malmö, Lund University, Jan Waldenströms gata 35, Skåne University Hospital, Malmö 205 02, Sweden; 2 Stanford Prevention Research Center, Stanford University School of Medicine, Stanford, CA, USA

**Keywords:** Hypertension, Yoga, Quality of life, Primary health care, Complementary therapies

## Abstract

**Background:**

Medical treatment of hypertension is not always sufficient to achieve blood pressure control. Despite this, previous studies on supplementary therapies, such as yoga, are relatively few. We investigated the effects of two yoga interventions on blood pressure and quality of life in patients in primary health care diagnosed with hypertension.

**Methods:**

Adult patients (age 20–80 years) with diagnosed hypertension were identified by an electronic chart search at a primary health care center in southern Sweden. In total, 83 subjects with blood pressure values of 120–179/≤109 mmHg at baseline were enrolled. At baseline, the patients underwent standardized blood pressure measurement at the health care center and they completed a questionnaire on self-rated quality of life (WHOQOL-BREF). There were three groups: 1) yoga class with yoga instructor (n = 28); 2) yoga at home (n = 28); and 3) a control group (n = 27). The participants were matched at the group level for systolic blood pressure. After 12 weeks of intervention, the assessments were performed again. At baseline a majority of the patients (92%) were on antihypertensive medication, and the patients were requested not to change their medication during the study.

**Results:**

The yoga class group showed no improvement in blood pressure or self-rated quality of life, while in the yoga at home group there was a decline in diastolic blood pressure of 4.4 mmHg (p < 0.05) compared to the control group. Moreover, the yoga at home group showed significant improvement in self-rated quality of life compared to the control group (p < 0.05).

**Conclusions:**

A short yoga program for the patient to practice at home seems to have an antihypertensive effect, as well as a positive effect on self-rated quality of life compared to controls. This implies that simple yoga exercises may be useful as a supplementary blood pressure therapy in addition to medical treatment when prescribed by primary care physicians.

**Trial registration:**

ClinicalTrials.gov (NCT01302535)

## Background

Hypertension is one of the most common diseases in the world, affecting approximately 26% of the adult population [1]. Persistent hypertension increases the risk of developing coronary heart disease, stroke and other cardiovascular diseases, such as heart failure [2,3]. Hypertension is a common diagnosis in primary health care and the societal costs of examination and treatment of hypertension and its consequences are considerable [4].

Although many antihypertensive drugs are available, fewer than one third of individuals in Europe who receive treatment reach their target blood pressure (BP) (140/90) [5]. Thus, additional strategies to normalize BP have been evaluated, e.g. lifestyle changes such as increased physical activity, weight loss, dietary improvement, stress management and reduced tobacco and alcohol intake [2].

Previous studies have also shown that yoga may reduce BP [6-9]. These studies showed significant reduction of systolic BP (SBP) of up to 6 mmHg and a significant reduction of diastolic BP (DBP) of up to 5 mmHg compared to baseline [6,8,9]. Whether these results are clinically significant remains an unanswered question.

If yoga has a BP lowering effect it may be useful as a supplementary therapy in addition to medical treatment.

Since some studies have shown that yoga positively impacts quality of life and subjective well-being [10-12], patients who regularly practice yoga may also experience better quality of life.

The novelty of the present study is that it was performed in a primary care setting where most patients with hypertension are treated.

The purpose of this matched controlled study was to determine the effects of yoga on BP and quality of life in patients in primary health care diagnosed with hypertension. Another aim was to investigate whether there is a difference in effect on BP and quality of life if yoga is practiced on a regular basis in a group led by a yoga instructor or if the patient practices a shorter yoga program individually at home.

## Methods

### Design

This study was a prospective three-arm single-center study of two types of yoga. It was designed as a matched controlled open clinical trial. BP measurement and other tests were carried out at baseline and after 12 weeks of intervention. There were two intervention groups and one control group. We matched the groups based on SBP since BP was the main outcome.

The design of the intervention in group one (yoga class group) has been successfully used previously in an inpatient care study (in Stockholm) of patients who have had myocardial infarction (unpublished data). We wanted to see if we could demonstrate this favorable effect in a primary care setting. The intervention for group two (yoga at home group) was designed in collaboration with the founder of the Institute for Medical Yoga (IMY) in Stockholm, Sweden.

Thus, we wanted to compare a yoga class approach with another, shorter and simpler yoga program (yoga at home) that would require less time and effort for the patient and the health care center.

### Patients and recruitment

Adult patients (age 20–80 years) at Svedala Primary Health Care Center in southern Sweden, diagnosed with hypertension, with BP 120–160/80–100 mmHg when last measured at the health care center (e.g. normal, normal high and grade 1 hypertension levels), were eligible for inclusion in the study. We did not want to select patients with extreme BP values, since these would probably be under medical adjustments.

Exclusion criteria were adjustments regarding hypertension treatment within the 4 weeks prior to the start of the study, expected inability to understand instructions about the yoga exercises, physical or mental incapacity to carry out yoga exercises and language difficulties/interpreter needs. Patients with systolic BP (SBP) ≥180 mmHg and/or diastolic BP (DBP) ≥110 mmHg or SBP <120 mmHg at baseline were also excluded. Our consideration regarding these limits was that it seemed unlikely to have any BP lowering effect on patients with SBP <120 mmHg. However, we often see DBP values <80 mmHg in our hypertensive patients. Regarding the upper limit, >180/110 mmHg, we thought it would be unethical to leave medication unchanged for twelve weeks.

Hence, patients with BP values of 120–179/≤109 mmHg at baseline were eligible for enrollment.

**Figure 1:**
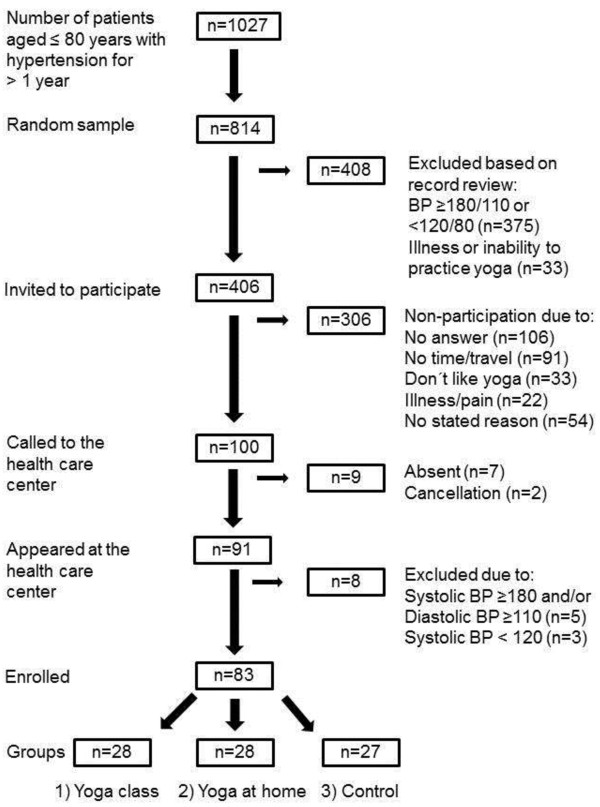
Flow chart outlining patient recruitment and the allocation of patients to the different groups.

As shown in Figure 1, 1027 patients were identified by the electronic chart search (hypertension diagnosis registered between 1 May 2008 and 31 January 2010). A random sample of 814 medical records (computer-generated randomization list) was reviewed regarding inclusion and exclusion criteria. The 406 eligible patients identified were invited by mail to participate in the study. Two weeks later they were contacted by telephone.

In March 2011, the patients who agreed to participate were invited to the health care center for baseline assessments. They were informed about the study and asked to provide written informed consent. BP was measured using automated devices. The participants completed a questionnaire on quality of life (WHOQOL-BREF [13]) and a health status and lifestyle survey designed for this study.

The participants were sorted numerically based on their SBP. They were then assigned, three at a time, to the three different groups in order to avoid large differences in SBP between the groups. This procedure yielded groups that were matched for SBP and it was performed by an independent statistician. There were no statistically significant differences in BP at baseline between the three groups.

The participants were requested not to change their medication during the study, and any change in medication was registered at follow-up.

After 12 weeks of intervention, all participants were assessed again. The patients who had practiced yoga answered questions about their experiences of the yoga.

### Interventions

The yoga practiced in the present study is a form of Kundalini yoga developed at the Institute for Medical Yoga (IMY) [14]. Kundalini yoga is relatively easy to perform compared with other forms of yoga and is suitable for all ages and levels of fitness. In most exercises the yoga movement is combined with powerful deep breathing. A typical Kundalini yoga class incorporates the following six elements: 1) tune-in with mantra, 2) warm-up or breathing exercises, 3) physical exercises or postures and breathing exercises, 4) deep relaxation, 5) meditation, and 6) tune-out with mantra.

### Intervention group 1 – yoga class with an instructor

Intervention group 1 (28 persons) was divided into three smaller groups, each consisting of 8–12 participants. Each group met once a week for 60 minutes at the health care center to practice yoga with a yoga instructor. The participants were encouraged to practice yoga for 30 minutes every day at home. In order to support their training at home, they received two instruction CDs (each approximately 50 minutes long) and two manuals. They were also given a yoga calendar in which to record when they did yoga.

The yoga classes comprised various yoga movements and positions, breathing techniques and meditation. The exercises were adapted for those who had trouble sitting on a yoga mat or had other difficulties in carrying out the exercises. The yoga was taught in a room, specially arranged for the purpose, with yoga mats, pillows, blankets and chairs. The entrance was separated from the health care center.

### Intervention group 2 – yoga at home

The participants in intervention group 2 (28 persons) were each given a doctor’s appointment (20 minutes) during which they received instructions for two yoga exercises, which they were encouraged to perform at home for a combined total of 15 minutes a day. The doctor was a study physician who was not a trained yoga instructor but was familiar with the yoga exercises and had received basic teacher instruction. The participants received one instruction CD (approximately 20 minutes long), a short manual and a yoga calendar.

The two yoga exercises were: 1) “Left nostril breathing” – deep breaths in and out through the left nostril while sitting or lying down, with the right nostril closed off by the right thumb or an earplug (about 11 minutes); and 2) “Spinal flex” – movement that alternates between flexing the spine forwards (arching) and relaxing the spine back in time with deep breaths while sitting in a chair (about 4 minutes).

In summary, the yoga class group had a more extensive yoga program, with several different exercises (including left nostril breathing and spinal flex). The educational material was provided by IMY [11].

### Group 3 – control

No changes were made for the participants in the control group (27 persons) (treatment as usual: treatment with the medication they were already taking and annual medical examination by the general practitioner).

### Outcome

The main outcome was BP at the end of the program and change in BP. The secondary outcome was self-rated quality of life.

### Measurements

#### ***Clinical data***

BP measurement was standardized in a sitting position, right arm, two readings (three readings when the first and second readings differed by >5 mmHg) [15], and was carried out by nurses using a validated BP monitor (Omron i-C10). BP was measured after 5–10 minutes of rest. Blood samples were collected at baseline for assessment of the following metabolic biomarkers: HbA1c, fasting plasma glucose and total cholesterol.

#### ***Self-reported data***

On their yoga calendars, the participants marked with a cross the dates they completed the yoga training. At follow-up, the patients submitted their yoga calendars. The information in the calendars was not controlled or questioned. As described above, the patients were encouraged to practice yoga once a day.

All patients who attended follow-up appointments were included in the analyses (as observed cases, OC). We also made calculations through which patients who did not perform yoga for at least 9/12 weeks or who changed their medication were excluded (per protocol set, PPS). This criterion (9/12 weeks) was set up together with the IMY founder, and it was not known to the patients.

The WHOQOL-BREF is a shorter version of the WHOQOL-100, containing 26 items which measure the following four domains: physical health, psychological health, social relationships and environment. There are also two items that are analyzed separately: overall perception of quality of life and overall perception of health.

The health status and lifestyle survey was designed for this study and is not validated. The survey contained questions regarding comorbidity for diabetes and CVD, smoking and drinking habits and physical activity.

### Statistical analysis

Assuming a mean treatment difference in SBP of 5 mmHg between the yoga at home and control groups, a standard deviation of 6 mmHg and a drop-out rate of 30%, 33 patients per group would have 80% power to detect a statistically significant difference at the 5% level using a two-sided test. The assumption about differences in SBP is based on a Swedish literature review [2].

The differences in mean change from baseline between the two yoga groups and the control group were investigated using a linear regression model with baseline BP as a continuous variable and treatment group as a categorical variable (ANCOVA). A corresponding model was used for quality of life (single items and domains). We studied the residuals to ensure that the normality assumption was not violated in a way that could affect the interpretation of the estimates. All tests were two-sided.

We further investigated whether other baseline characteristics (age, sex and BMI) could influence the outcome using the same linear regression model as above and including the baseline characteristic of interest. However, the study was not sufficiently powered to do subgroup analyses or to detect statistically significant differences for other covariates.

Version 9.2 of the SAS System for Windows was used in the statistical analyses.

### Ethical aspects

The study conforms to the principles outlined in the Declaration of Helsinki and was approved by the Regional Ethical Review Board in Lund, Sweden (2010/728). The study was registered at ClinicalTrials.gov (NCT01302535).

## Results

The baseline characteristics of the patients in the three groups are presented in Table 1. There was a predominance of women in all three groups. The majority of the patients were overweight (BMI >25 kg/m^2^) and 92% were on antihypertensive medication (Table 1). At baseline, 37% of the patients were well controlled (≤140/90 mmHg). The most common single treatment was beta blockers (BB, 13%). The study was not powered to detect difference or change in morbidity, but there were no significant differences between the groups regarding prevalence of diabetes or cardiovascular disease at baseline. This was also the case for the results regarding physical activity from the lifestyle survey.

**Table 1:** Baseline characteristics

	**Intervention group 1**	**Intervention group 2**	**Group 3**
	**Yoga class group**	**Yoga at home group**	**Control group**
	**n = 28**	**n = 28**	**n = 27**
	**Mean (SD)**	**Mean (SD)**	**Mean (SD)**
Age in years	66.2 (7.7)	64.0 (10.3)	60.8 (11.0)
% female	67.9	71.4	59.3
BMI (kg/m^2^)	29.7 (7.0)	28.5 (7.3)	28.8 (4.0)
SBP (mmHg)	143.8 (14.9)	143.6 (14.2)	144.3 (14.5)
DBP (mmHg)	89.0 (7.6)	88.4 (6.2)	89.8 (7.3)
On medication (%)	96.4	92.9	85.2
Well controlled (%)§	39.3	35.7	37.0
Drugs§§	2.0 (1.1)	1.8 (1.0)	1.4 (1.0)
ACEI (n)	10	6	8
ARB (n)	7	5	5
Thiazides (n)	13	17	8
CCB (n)	10	10	4
BB (n)	17	11	13
Loop diuretics (n)	0	1	1
FP glucose (mmol/L)	5.5 (0.8)	5.4 (1.6)	5.9 (2.3)
HbA1c (mmol/mol)	40.9 (10.5)	40.0 (8.5)	39.6 (10.1)
Cholesterol (mmol/L)	5.2 (1.0)	5.4 (1.1)	5.3 (1.2)
WHO 1†	3.59 (0.8)	4.07 (0.7)*	3.96 (0.7)
WHO 2‡	3.04 (0.9)	3.61 (0.9)*	3.31 (0.7)

§BP ≤140/90 mmHg at baseline.

§§Number of different antihypertensive drugs.

†WHO 1: How would you rate your quality of life? Very poor (1), poor (2), neither poor nor good (3), good (4), very good (5).

‡WHO 2: How satisfied are you with your health? Very dissatisfied (1), dissatisfied (2), neither satisfied nor dissatisfied (3), satisfied (4), very satisfied (5).

*Significant difference compared to control (p < 0.05).

SD, standard deviation; BMI, body mass index; SBP, systolic blood pressure; DBP, diastolic blood pressure; ACEI, angiotensin-converting enzyme inhibitor; ARB, angiotensin receptor blocker; CCB, calcium receptor blocker; BB, beta blocker.

**Figure 2:**
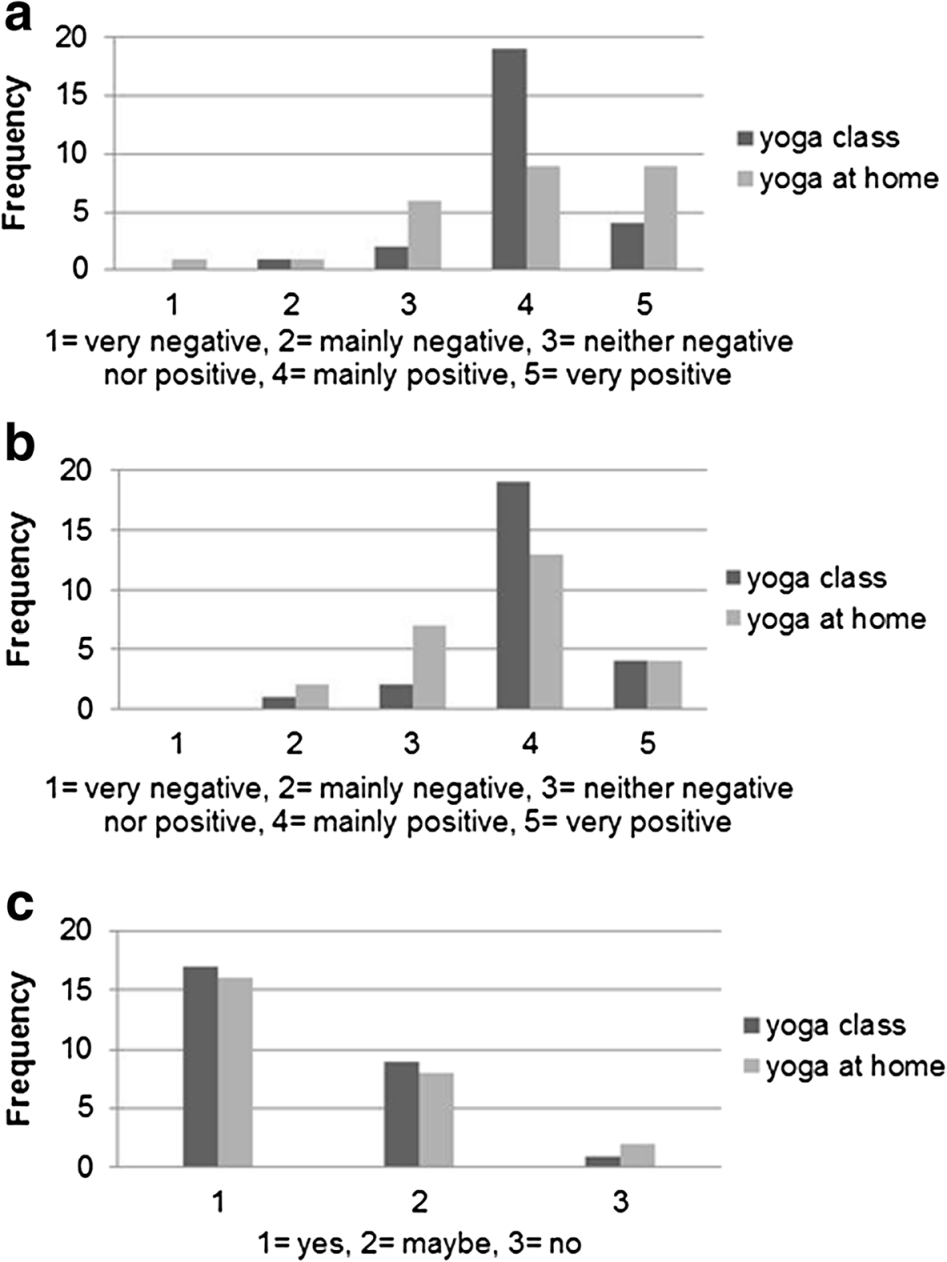
**Patients’ views on the yoga practice.** Patients in the yoga at home and yoga class groups were asked three questions about their experiences of the yoga. **a**. How did you experience the yoga practice emotionally? **b**. How did you experience the yoga practice physically? **c**. Will you continue practicing yoga after this study?

Table 2 shows changes in SBP and DBP in the three groups. No significant differences in change in SBP from baseline between the yoga groups and the control group were detected. However, the improvement in DBP for the yoga at home group was significantly greater than that for the control group (-4.4 ± 1.6 vs. 0.8 ± 1.6 mmHg; OC, p < 0.05). The yoga at home group also showed significant improvements in self-rated quality of life compared to the control group (0.29 ± 0.13 vs. 0.06 ± 0.13 for WHOQOL, Item 1; OC, p < 0.05) (Table 3). There were no significant differences in change in DBP or self-rated quality of life between the yoga class and the control groups. Further analyses of the WHOQOL domains did not demonstrate any significant changes in any group (data not shown). Compliance with yoga practice (number of yoga sessions) was lower in the yoga class group than in the yoga at home group (Table 2). However, the average total time spent on yoga practice was higher in the yoga class group (about 24 hours vs. 16 hours). Both yoga groups reported mainly positive experiences concerning the yoga practice (Figure 2a-b), and only three participants stated that they would not continue with yoga after the study (Figure 2c).

**Table 2:** BP after intervention and adjusted mean change in BP

	**Intervention group 1**		**Intervention group 2**		**Group 3**	
	**Yoga class group**		**Yoga at home group**		**Control group**	
	**OC**	**PPS**	**OC**	**PPS**	**OC**	**PPS**
	**n = 28**	**n = 21**	**n = 26**	**n = 20**	**n = 26**	**n = 23**
	**Mean (SE)**	**Mean (SE)**	**Mean (SE)**	**Mean (SE)**	**Mean (SE)**	**Mean (SE)**
**SBP** (mmHg)	144.1 (2.6)	144.3 (3.1)	137.0 (2.7)	138.4 (3.2)	141.5 (2.7)	142.6 (3.0)
Change from baseline	0.3 (2.6)	-0.2 (3.1)	-6.8 (2.7)	-6.1 (3.2)	-2.3 (2.7)	-1.9 (3.0)
P-value	0.917	0.954	0.013**	0.061	0.381	0.527
Difference vs. control	2.6 (3.7)	1.7 (4.3)	-4.4 (3.8)	-4.2 (4.4)		
P-value	0.482	0.693	0.241	0.341		
**DBP** (mmHg)	89.4 (1.6)	89.5 (1.9)	84.7 (1.6)	85.3 (1.9)	89.9 (1.6)	90.2 (1.8)
Change from baseline	0.2 (1.6)	0.3 (1.9)	-4.4 (1.6)	-3.9 (1.9)	0.8 (1.6)	1.01 (1.8)
P-value	0.889	0.890	0.008**	0.045**	0.612	0.571
Difference vs. control	-0.6 (2.5)	-0.8 (2.6)	-5.2 (2.3)	-4.9 (2.6)		
P-value	0.794	0.771	0.025*	0.064		
Yoga sessions during intervention†	47.2	52.7	63.9	72.6		

*Significant difference compared to control (p < 0.05).

**Significant change from baseline (p < 0.05).

†According to yoga calendar (yoga class sessions and yoga at home sessions).

OC, observed cases; PPS, per protocol set; SE, standard error of the mean; SBP, systolic blood pressure; DBP, diastolic blood pressure.

The PPS consists of all patients who (1) practiced yoga at least once a week for nine weeks or more and (2) had no change in medication during the study period.

**Table 3:** Self-rated quality of life and self-rated health after intervention

	**Intervention group 1**	**Intervention group 2**	**Group 3**
	**Yoga class group**	**Yoga at home group**	**Control group**
	**OC, n = 28, Mean (SE)**	**OC, n = 26, Mean (SE)**	**OC, n = 26, Mean (SE)**
**WHO1**†			
Average score	3.98 (0.09)	4.21 (0.09)	3.92 (0.09)
Change from baseline	0.12 (0.09)	0.35 (0.09)	0.06 (0.09)
Difference vs. control	0.06 (0.13)	0.29 (0.13)	
P-value	0.640	0.027*	
**WHO2**†			
Average score	3.44 (0.11)	3.68 (0.12)	3.41 (0.12)
Change from baseline	0.15 (0.11)	0.39 (1.12)	0.11 (0.12)
Difference vs. control	0.04 (0.16)	0.28 (0.16)	
P-value	0.826	0.099	

†For questions (WHOQOL, Items 1–2), refer to Table 1.

*Significant difference compared to the other groups (p < 0.05) (ANOVA).

OC, observed cases; SE, standard error of the mean.

SBP decreased on average by 5.4 mmHg (p = 0.096) more in women than in men, after correction for baseline BP and treatment (all groups, data not shown). The difference between the decreases in DBP in men and women was 2.2 mmHg (p = 0.27) (data not shown). However, the study was not sufficiently powered to detect any statistically significant differences regarding sex.

## Discussion

The present study was conducted to determine the effects of yoga on BP and quality of life in patients in primary health care. Our results demonstrated a significant reduction in DBP in the patients who practiced yoga at home compared to the control group (p < 0.05). The yoga at home group also showed a greater improvement in quality of life than the control group (p < 0.05). Patients who practiced yoga in a group with an instructor, however, did not experience significant improvements in BP or self-rated quality of life compared to the control group. Only three of the 83 participants failed to attend the follow-up appointment. This means that a sufficient number of patients completed the study according to the power calculation.

The results imply that simple yoga exercises may be useful as a supplementary BP therapy in addition to medical treatment when prescribed by primary care physicians.

It is well known that physical activity has a BP lowering effect. For those patients who are not able or willing to do demanding exercise, an easy yoga program could be an alternative.

It is interesting to note that a relatively small effort for the health care center (in terms of number of visits) had the best effect on BP and quality of life.

The present study contributes to yoga-hypertension research by examining the effects of yoga in a primary health care setting, where most patients with hypertension are treated. The shorter intervention (yoga at home) can easily be taught to the patient by his or her own doctor at the health care center.

Previous studies have shown that yoga reduces BP [6-9,16-20]. However, the yoga intervention design varied among these studies and the length of the intervention ranged from 3 to 20 weeks, making it difficult to compare the interventions in terms of effectiveness. Furthermore, some of the studies combined the yoga treatment with other measures, such as changes in diet [19,20].

One of four invited patients (25%) chose to participate in the study. These patients were probably open-minded about complementary and alternative therapy. In view of this selection bias, the results of the study are probably not applicable to all patients in primary care with hypertension. However, this is the case in most other comparable yoga studies. As reported, the yoga at home patients rated their quality of life at baseline higher than the other groups. This fact could indicate a higher motivation among these patients to try something new to further improve their quality of life. On the other hand, one could argue that it is more difficult to improve quality of life when starting from a higher level. Additionally, the fact that the yoga at home group had an early private appointment with a study physician may in itself have had a positive effect on the results [21]. Patients probably adhere more to a doctor’s advice about yoga when yoga is used as a supplementary therapy. However, the patients in the yoga at home group only met the doctor for 20 minutes, while the yoga class group patients met their instructor for 12 hours during the intervention period.

It is unclear why the yoga class group did not have any reduction in BP. One possible explanation lies in the yoga exercises the two groups performed at home. The yoga class group had a more advanced yoga program than the yoga at home group. This may have contributed to the fact that the yoga class group participants performed fewer yoga sessions at home during the intervention than the yoga at home participants. The number of sessions may have influenced the result, but the yoga class group patients spent on average about 50% more time doing yoga than the yoga at home group patients. There might have been additional barriers to the yoga class group members than the advanced exercises, such as travelling to the health care center each week. Being in a class environment with other patients could also make some people feel insecure and uncomfortable. However, one could also argue that these barriers would be balanced by a rewarding interaction with the instructor and other group members.

According to a Swedish literature review, the mean reduction of BP from an antihypertensive drug is 10/5 mmHg, when used alone [2]. The effect of an additional drug is mostly lower. In view of this fact, the mean reduction of DBP of 4.4 mmHg, shows that the effect of the short yoga program could be of clinical relevance and interest when used as a supplement to other treatment.

A weakness of the study concerns the self-reported data (yoga calendar), which is a problem in all studies of this kind. In general, studies on supplementary intervention are difficult to perform, but none the less it is an important study since yoga is increasingly popular and practiced by many people.

The fact that the proportion of women in the different groups varied, with the highest proportion of women in the yoga at home group, may have influenced the results to some extent, since women tended to have a greater SBP-lowering response to yoga than men. However, the differences in age and gender between the groups were not significant.

The participants were matched for SBP at study start. They were not matched regarding the number of medicines. The yoga class group patients had on average more antihypertensive drugs than the yoga at home and control group patients. The differences between the groups were not significant, but more medicines could indicate more severe hypertension.

A 24-hour ambulatory BP measurement would be a more accurate method to measure BP over time, but this was not possible in our study. Moreover, even studies on the effects of medicine on BP are usually not made with 24-hour ambulatory BP measurement.

Randomized allocation is superior to matching in most studies, and this is a limitation of the study. Our rationale for matching the groups was that we wanted to ensure similar SBP values at baseline.

## Conclusion

A short yoga program for patients to practice at home seems to have an antihypertensive effect, as well as a positive effect on self-rated quality of life. This implies that simple yoga exercises may be useful as a supplementary BP therapy in addition to medical treatment when prescribed by primary care physicians. One could also speculate as to whether this in the long run could influence medicine intake, side effects and drug costs. However, larger, randomized controlled studies are needed to confirm the antihypertensive effect of yoga and to identify the groups of patients that will benefit most from yoga-based treatment. We also need to study the long-term effects of yogic treatment on hypertension.

## Abbreviations

ACEI: Angiotensin-converting enzyme; ARB: Angiotensin receptor blocker; BB: Beta blocker; BMI: Body mass index; BP: Blood pressure; CCB: Calcium channel blocker; DBP: Diastolic blood pressure; FP: Fasting plasma; IMY: Institute for medical yoga; OC: Observed cases; PPS: Per protocol set; SBP: Systolic blood pressure.

## Competing interests

The authors declare that they have no competing interests.

## Authors’ contributions

MW is the principal investigator. MW and PM designed the study in collaboration with KS. SLL conducted the statistical analysis of the material. MW drafted the manuscript. PM, SLL and KS revised the manuscript critically. All authors read and approved the final manuscript.

## Pre-publication history

The pre-publication history for this paper can be accessed here:

http://www.biomedcentral.com/1471-2261/13/111/prepub
